# Only a Minority of Thrombectomy Candidates Are Admitted During Night Shift: A Rationale for Diurnal Stroke Care Planning

**DOI:** 10.3389/fneur.2020.573381

**Published:** 2020-09-30

**Authors:** Björn Reuter, Christian Stock, Matthias Ungerer, Sonja Hyrenbach, Ingo Bruder, Peter A. Ringleb, Rolf Kern, Christoph Gumbinger

**Affiliations:** ^1^Department of Neurology and Geriatrics, Helios Klinik Müllheim, Müllheim, Germany; ^2^Institute of Medical Biometry and Informatics, University of Heidelberg, Heidelberg, Germany; ^3^Department of Neurology, Heidelberg University, Heidelberg, Germany; ^4^Office for Quality Assurance in Health Care System Baden-Württemberg LLC (QiG BW GmbH), Stuttgart, Germany; ^5^Department of Neurology, Klinikum Kempten, Kempten, Germany

**Keywords:** ischemic stroke, daytime, stroke severity, thrombectomy, stroke care planning

## Abstract

**Background:** Widespread quick access to mechanical thrombectomy (MT) for acute ischemic stroke (AIS) is one of the main challenges in stroke care. It is unclear if newly established MT units are required 24 h/7 d. We explored the diurnal admission rate of patients with AIS potentially eligible for MT to provide a basis for discussion of daytime-adapted stroke care concepts.

**Methods:** Data collected from the Baden-Württemberg Stroke Registry in Germany were assessed (2008–2012). We analyzed the admission rate of patients with AIS stratified by the National Institutes of Health Stroke Scale (NIHSS) score at admission in 3-h intervals. An NIHSS score ≥10 was considered a predictor of large vessel occlusion. The average annual admission number of patients with severe AIS were stratified by stroke service level and calculated for a three-shift model and working/non-working hours.

**Results:** Of 91,864, 22,527 (21%) presented with an NIHSS score ≥10. The average admission rates per year for a hospital without Stroke Unit (SU), with a local SU, with a regional SU and a stroke center were 8, 52, 90 and 178, respectively. Approximately 61% were admitted during working hours, 54% in the early shift, 36% in the late shift and 10% in the night shift.

**Conclusions:** A two-shift model, excluding the night shift, would cover 90% of the patients with severe AIS. A model with coverage during working hours would miss ~40% of the patients with severe AIS. To achieve a quick and area-wide MT, it seems preferable for newly implemented MT-units to offer MT in a two-shift model at a minimum.

## Introduction

Based on positive results from randomized controlled trials published in 2015, the implementation of mechanical thrombectomy (MT) for selected patients with acute ischemic stroke (AIS) was started immediately (1, 2). At this time, the landscape of acute stroke therapy in Europe and abroad were dominated by treatment with intravenous thrombolysis (IVT) on stroke units (SU) and consequently needed adaptation (3). Patients with AIS and large vessel occlusion (LVO) now require quick transfer to a hospital providing MT, usually using a “drip and ship” approach (4).

In the past years, widespread implementation of time-efficient endovascular stroke care delivery has made significant progress, however, it remains a challenge particularly in rural areas (5).

In the German federal state of Baden-Württemberg (BW), acute stroke care is provided at 13 stroke centers offering maximum care, 20 hospitals with regional SUs and 18 hospitals with local SUs^1^. Together they cover an area of ~35,750 km^2^ and 11 million inhabitants^1^. Stroke centers are required to offer MT 24 h/7 d. In recent years, hospitals with regional SUs have started to provide MT either 24 h/7 d or during regular working hours, despite the preferred 24 h/7 d coverage by the German Stroke Society (6). Healthcare policy in Baden Württemberg currently undertakes extensive efforts to close gaps in coverage^2^.

Setting up new MT units and providing MT 24 h/7 d is demanding in terms of skilled professionals and high costs involved (2, 3). As admission time of patients with AIS is not uniformly distributed throughout the day, e.g., admission rates are lower during nighttime, 24 h/7 d coverage by every provider of MT may not be optimal for both the individual hospital and healthcare system. In this study, we investigated the potential loss in coverage of MT for eligible patients if this therapy was not provided 24 h/7 d (7).

## Materials and Methods

We performed a retrospective database study using the BW stroke registry in Germany. The study was approved by the ethics committee of the Medical Faculty of the University of Heidelberg.

### Setting, Eligibility Criteria and Study Size

A comprehensive description of the BW stroke registry is provided elsewhere (8). In 2004, the federal state of BW implemented a three-level stroke care concept with stroke centers offering the full spectrum of modern stroke treatment, regional SUs and local SUs (the latter often using tele-neurology) to provide close to home stroke care with short transportation times. Quality of care was monitored by the Office for Quality Assurance in Hospitals (QiG BW). Pseudonymised data on all stroke patients hospitalized within 7 days after stroke onset and a minimum 18 years of age were collected.

We analyzed data covering a period of 5 years from January 1, 2008, to December 31, 2012, i.e., before the implementation of MT in clinical routine in 2015. The preceding years 2013 and 2014 were also not included into our analysis to avoid bias due to transportation to stroke centers, which increasingly started to offer endovascular therapy based on individual decision making. All registered patients with discharge diagnosis AIS were included in the analysis. Missing data on the National Institutes of Stroke Scale (NIHSS) score at admission was considered as an exclusion criterion.

### Variables

In a recent meta-analysis, the NIHSS score at admission was identified as a predictor of large vessel occlusion (9). An NIHSS score ≥10 would equally balance sensitivity and specificity with 73 and 74%, respectively. We thus regarded a threshold of NIHSS ≥10 suitable for our analysis (9).

The following data from the BW stroke registry were used from electronic patient records: patient demographic data, stroke onset time, hospital admission time and level of hospital care. AIS severity at admission was categorized using the NIHSS score ≤3 (mild AIS), 4–9 (moderate AIS) and ≥10 (severe AIS). For calculation of overall admission rates, daytime was stratified into 3-h intervals (00:00–03:00, 03:01–06:00, 21:01–23:59). Data were then stratified by the hospital level of stroke care (stroke centers, hospitals with regional stroke units (SUs), local SUs and without SUs), and either working vs. non-working hours (08:00–16:59, 17:00–07:59) or according to a three-shift system (07:00–14:59, 15:00–22:59, 23:00–06:59) to calculate the respective hospital admittance rates.

### Statistical Analysis

We used standard descriptive statistics to explore the admission by daytime. Data are shown as absolute numbers and/or percentages. Multivariable Poisson regression models were used to assess potential associations between hospital admission time and NIHSS score at admission. To facilitate interpretation, we estimated the average annual admission rates per hospital, conditional on its stroke care level (assuming a similar distribution of patients between hospitals of the same stroke care level). All statistical tests were two-sided. As no adjustment for multiplicity was performed, the *p*-values needed to be interpreted descriptively. The analyses were carried out using SAS 9.4 (SAS Institute Inc., Cary, NC, USA).

### Availability of Data and Materials

The source data of the stroke registry are not publicly available due to privacy regulations in Germany. The study protocol used for this analysis is available from the corresponding author.

## Results

### Study Population

Overall, 109,530 patients with AIS were identified from the database (Table 1, baseline characteristics), and 15.9% of the patients were excluded from the analysis due to missing NIHSS scores at admission. The median age was 76 years and the sex ratio was balanced with 49% of female patients. Of the remaining 91,864 patients with AIS, 43% presented with an NIHSS score ≤3, 33% with an NIHSS score ranging 4–9 and 24% with an NIHSS score ≥10. In line with the previous findings, an inverse association between stroke severity and onset to door-time became evident (median, NIHSS ≥10: 246 min, 4–9: 389 min, ≤3: 503 min) (10). Patients excluded from this analysis had a longer onset-to-door time (median 630 min) than the subgroup of patients with mild stroke.

**Table 1:** Characteristics of the study Population.

**Variable**	
Patients, N	109,530
Age, median (IQR)	76 (68, 83)
Female sex, N (%)	54 250 (49)
NIHSS score at admission^*^	
≤ 3, N (%)	39 202 (43)
median (mean)	2 (1.6)
4–9, N (%)	30 135 (33)
median (mean)	6 (5.8)
≥10, N (%)	22 527 (25)
median (mean)	16 (16.8)
**Median onset to door-time in minutes (mean)**	
NIHSS score ≤3	503 (1,348)
NIHSS score 4–9	389 (1,045)
NIHSS score ≥10	246 (723)
NIHSS scores missing	630 (1,553)
**Level of care**	
Stroke center, N (%)	26 794 (24)
Regional SU, N (%)	19 723 (18)
Local SU, N (%)	40 864 (37)
No SU, N (%)	22 149 (20)

* *The NIHSS score at admission was missing for N = 17,666 patients or 16% of the study population. Abbreviations: IQR, interquartile range; NIHSS, National Institutes of Health Stroke Scale; SU, stroke unit*.

**Figure 1:**
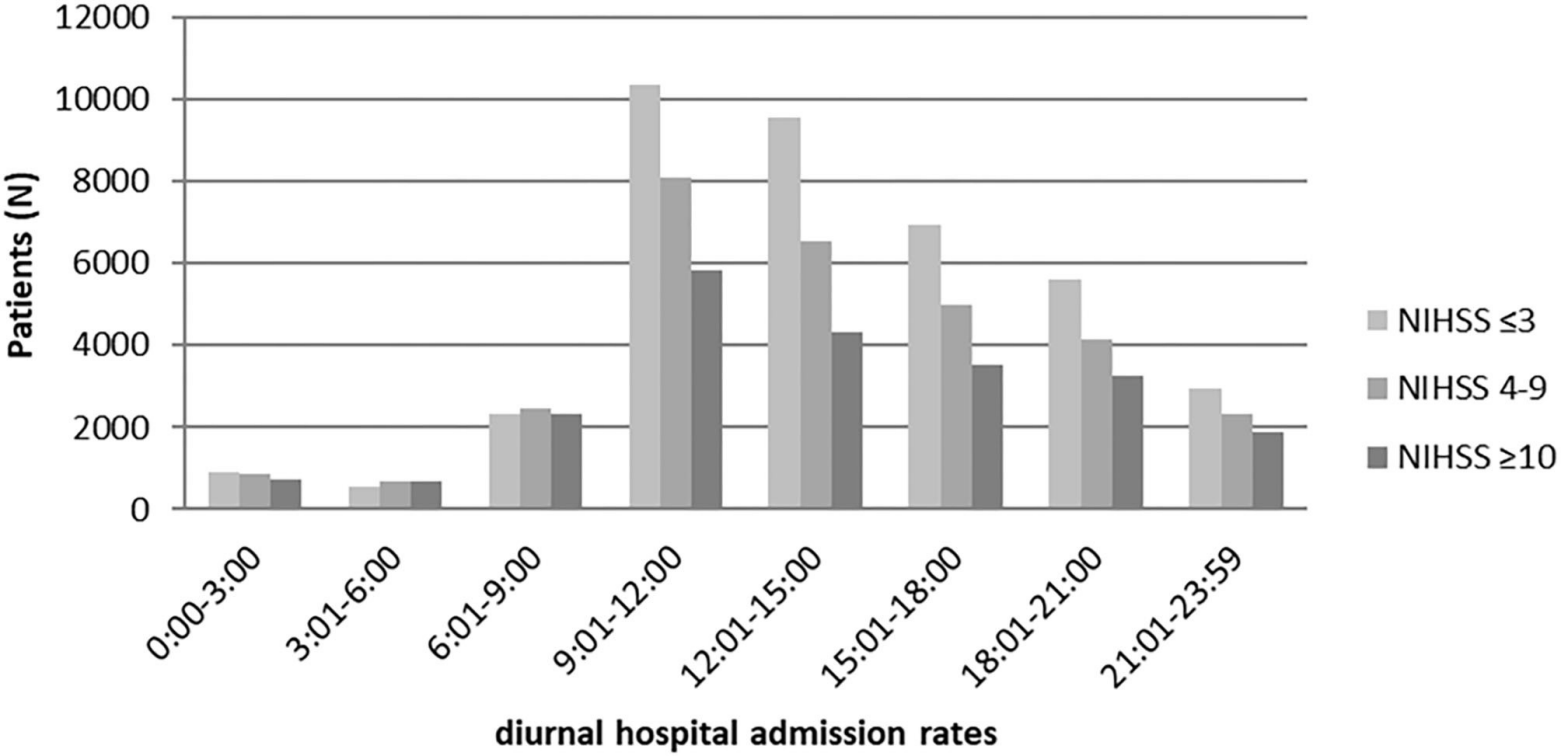
Diurnal hospital admission numbers stratified by stroke severity.

Stroke admission rates have a strong morning peak, which was observed regardless of stroke severity (Figure 1, Supplementary Table 1) (7). Absolute admission numbers were highest between 09:01 and 12:00 and lowest between 03:01 and 06:00 for all subgroups. Multivariable Poisson regression analyses suggested that patients with an NHSS score ≥10 had the most uniform diurnal admission rate of all patients (Supplementary Table 2).

### Admission of Severely Affected Patients With AIS

Of the 22,527 patients with an NIHSS score ≥10, 28% were admitted to stroke centers, 16% to hospitals with regional SUs, 39% to hospitals with local SUs and 17% to hospitals without a SU (Table 2).

**Table 2:** Admission of AIS patients with NIHSS ≥10 stratified by the level of care.

**Stroke service level**	**Admission numbers and admission rate, *N* (%)**	**Average annual admission rate per hospital, *N***
Stroke centers (*N* = 7)	6,229 (28)	178
Hospitals with regional SU (*N* = 8)	3,589 (16)	90
Hospitals with local SU (*N* = 34)	8,860 (39)	52
Hospitals w/o SU (*N* = 93)	3,849 (17)	8

*In total N = 22,527 patients with NIHSS ≥10 were admitted. NIHSS, National Institutes of Health Stroke Scale; SU, stroke unit*.

The estimated average annual admission rate per hospital-type were *N* = 8 patients with AIS with an NIHSS score ≥10 to a hospital without a SU, *N* = 52 patients to a local SU, *N* = 90 patients to a regional SU, and *N* = 178 patients to a stroke center.

We further calculated the admission numbers for common working time models, i.e., working vs. non-working hours and according to a three-shift system (Table 3, data is presented as average annual numbers). Of these patients, 61% (*N* = 2,764) were admitted during working hours compared to 39% (*N* = 1,742) during non-working hours. In a hypothesized three-shift system, 54% (*N* = 2,420) were admitted in the early shift, 36% (*N* = 1,632) in the late shift and 10% (*N* = 454) in the night shift. We observed only minor differences between the hospital levels with slightly higher admission rates to stroke centers during non-working hours or the late and night shift compared to the other hospital levels. A threshold NIHSS of ≥6, as recommended MT criterion in current stroke guidelines, would allow a higher sensitivity of 87% and lower specificity of 52% to identify LVO (9, 11, 12). Out of *N* = 91,864 AIS patients *N* = 37,703 (41%) presented with NIHSS ≥6. Absolute numbers were constantly ~ 1.66 times higher compared to a threshold NIHSS ≥10, and percentages in further subgroup analyses were similar.

**Table 3:** Average admission rate of AIS patients with NIHSS ≥10 per hospital and year.

	**Daytime of hospital admission**, ***N*** **(%)**				
**Stroke service level**	**Working hours**	**Non-working hours**	**Early shift**	**Late shift**	**Night shift**
	**08:00–16:59**	**17:00–07:59**	**07:00–14:59**	**15:00–22:59**	**23:00–06:59**
Stroke center	103 (58)	75 (42)	89 (50)	67 (38)	22 (12)
Hospital with regional SU	55 (61)	35 (39)	49 (54)	32 (36)	9 (10)
Hospital with local SU	33 (62)	20 (38)	29 (55)	19 (36)	5 (9)
Hospital w/o SU	5 (63)	3 (38)	5 (63)	2 (25)	1 (13)
Overall annual numbers	2,764 (61)	1,742 (39)	2,420 (54)	1,632 (36)	454 (10)

*Daytime is stratified into working vs. non-working hours and into a three-shift system. Presented are the average annual numbers of patients and percentages per hospital within the respective time intervals. Averages are calculated from 7 stroke centers, 8 regional SUs, 34 local SUs, and 93 hospitals without a SU. NIHSS, National Institutes of Health Stroke Scale; SU, stroke unit*.

## Discussion

Diurnal emergency calls and hospital admission rates for cerebrovascular and cardiovascular disease follow a distinct pattern with a strong morning peak and generally higher rates during daytime compared to nighttime hours (7, 13–15). In our analysis of 91,864 patients with AIS, the average annual admission rate for patients with severe AIS ranged from *N* = 8 at hospitals without a SU to *N* = 178 at stroke centers. Even though the admission rate of those with severe AIS was found to be more balanced over the daytime compared to patients with mild or moderate AIS, the vast majority (90%) were admitted during the early and late shifts of a three-shift model (07:00–14:59, 15:00–22:59, 23:00–06:59); and 61% of patients with severe stroke were hospitalized during working hours. The average annual hospital admission rate during the night shift further demonstrates the low frequency, with *N* = 5 patients being admitted in this time interval to local SUs, *N* = 9 to regional SUs and *N* = 22 to stroke centers. A threshold NIHSS of ≥6 would increase admissions roughly by the factor 1.66, while the proportions and thus implications remained equal.

### Discussion of Results for Clinical Practice

Since 2015, the selection criteria for MT candidates with LVO have frequently been adjusted. In 2018, the DAWN and DEFUSE-3 trials demonstrated that MT was successfully applicable up to 24 h after stroke onset (16–18). Further trials investigated borderline LVO patients, such as large ischemic cores or distal occlusions (19, 20). The number of MT procedures is expected to increase further. This ongoing evolution of acute stroke recanalization strategies will make new concepts of pre- and in-hospital care delivery necessary (21–23). It seems reasonable to consider the diurnal variation of the stroke for future implementation strategies of MT (7, 13).

In recent years, hospitals in Germany with regional SUs or even tele-stroke units have started to offer MT, with some of them not covering the entire day (24). Based on our data, offering MT outside stroke centers during working hours would cover 62% of the admitted potential MT candidates with severe stroke. Furthermore, nearly 40% of the patients would still need secondary transfer for MT. The rate of coverage would increase to 90% with a two-shift system ranging from 07:00–22:59. The remaining 10% during the night shift represents *N* = 2,269 patients with a relevant probability of large vessel occlusion being admitted over 5 years, or ~9 patients per week. These data suggest that the current 13 stroke centers in BW will not encounter capacity issues while treating these patients.

It might be reasonable that some SUs do not offer MT during the nighttime as an implementation strategy if they cannot provide 24 h/7 d coverage. The herein suggested strategy might be, in our opinion, an efficient attempt to avoid treatment delays by primary or secondary transportation to hospitals with MT capacities for a large proportion of patients with AIS at high risk of severe neurological disability in due consideration of limited qualified personnel resources. Moreover, this would unburden both the emergency medical services through less and shorter transportation times and stroke centers. As this model needs careful adjustment of treatment and transportation capacities, it should be implemented within stroke networks (25). In our opinion, providing MT intermittently should only be an implementation strategy.

### Limitations

In particular, two limitations should be considered while interpreting our results. Firstly, the threshold NIHSS ≥10 serves as a proxy to estimate possible MT candidates. The NIHSS is a common assessment tool to estimate AIS severity in in-hospital settings and stroke registries (26, 27). An NIHSS ≥10 was identified as the most balanced threshold to predict LVO with a sensitivity of 73% and specificity of 74% (9). Thus, the NIHSS score is a frequently used prediction instrument for LVO (28, 29).

Secondly, ~15.9% of the patients with AIS were excluded from the analysis because the NIHSS score at admission was missing. The median onset-to-door time suggests that these patients mainly suffered minor AIS and hence do not alter the core results of our analysis. Their median onset to door-time of 630 min was found to be longer than the onset to door-time of our NIHSS ≤3 subgroup; therefore, it is unlikely that a relevant proportion of patients were candidates for thrombectomy.

## Conclusion

When stratified by stroke severity, patients with severe AIS (based on NIHSS score ≥10) demonstrated a more balanced diurnal admission rate compared to patients with mild or moderate AIS. Nevertheless, a majority of 90% are admitted during the daytime and evening hours and the numbers admitted during the night shift are very low. At hospitals with regional SUs, an endovascular service during working hours (08:00–16:59) would cover 62% of the patients with severe AIS, which would reach up to 90% based on a two-shift system ranging from 07:00–22:59. The data may help to optimize implementation strategies for MT, with the consideration of starting MT with a two-shift model. Furthermore, our results may help to adjust emergency medical services transportation capacities within stroke networks.

## Data Availability Statement

The source data of the stroke registry are not publicly available due to privacy regulations in Germany. The study plan and statistical design used in this analysis are available from the corresponding author. Requests to access these datasets should be directed to christoph.gumbinger@med.uni-heidelberg.de.

## Ethics Statement

The studies involving human participants were reviewed and approved by the ethics committee of the Medical Faculty of the University of Heidelberg (S339-2012). Written informed consent for participation was not required for this study in accordance with the national legislation and the institutional requirements.

## Author Contributions

BR, CS, and CG designed the study. BR analyzed data and wrote the paper. CG and CS designed and performed the statistical analysis, analyzed data, and revised the paper. MU was involved in the interpretation of data and revised the paper. RK and PR were involved in the planning of analysis and the interpretation of data. SH and IB prepared the source data. The Stroke Working Group of the federal state of Baden-Wuerttemberg was involved in the interpretation of data and revised the paper. All authors agreed to the final version of the manuscript.

## Conflict of Interest

SH and IB were employed by the company QiG BW GmbH. RK has received speaker's honoraria from Boehringer Ingelheim, Bayer, Pfizer/BMS, Novartis and Daiichi Sankyo, unrelated to this study. PR has received lecture fees and travel compensation from Boehringer-Ingelheim, Ferrer, Paion, Bayer, and Sanofi, unrelated to this study. CS is now a full-time employee of Boehringer Ingelheim Pharma GmbH & Co. The company had no role in the design, analysis or interpretation of this study. Views expressed in this article are those of the authors and do not reflect those of Boehringer Ingelheim Pharma GmbH & Co. CG is the head of the commission telestroke service of the German Stroke Society (DSG). The remaining authors declare that the research was conducted in the absence of any commercial or financial relationships that could be construed as a potential conflict of interest.

## Footnotes

^1^*Arbeitsgemeinschaft Schlaganfallstationen Baden-Württemberg e.V*, Avaliable online at: http://www.schlaganfallstationen-bw.de/

^2^*Schlaganfallkonzeption Baden-Württemberg*. Avaliable online at: https://sozialministerium.baden-wuerttemberg.de/fileadmin/redaktion/m-sm/intern/downloads/Publikationen/Schlaganfallkonzeption_BW_Maerz_2019.pdf
