# Supplementary material for: Only a Minority of Thrombectomy Candidates Are Admitted During Night Shift: A Rationale for Diurnal Stroke Care Planning
Source: Front Neurol. 2020 Sep 30;11:573381. doi: 10.3389/fneur.2020.573381 (PMC7555607; doi:10.3389/fneur.2020.573381)
Supplement: Supplementary file 1 [file Table_1.DOCX]

| **Supplemental Table S1.** Daytime of hospital admission stratified by stroke severity | | | | | | | | | | |
| --- | --- | --- | --- | --- | --- | --- | --- | --- | --- | --- |
|  |  | **Daytime of hospital admission** | | | | | | | |  |
|  |  | 0:00–3:00 | 3:01–6:00 | 6:01–9:00 | 9:01–12:00 | 12:01–15:00 | 15:01–18:00 | 18:01–21:00 | 21:01–23:59 | overall |
| **NIHSS Score** |  |  |  |  |  |  |  |  |  |  |
| ≤3 | *N* | 908 | 572 | 2326 | 10362 | 9541 | 6959 | 5596 | 2938 | 39202 |
|  | *% (subgroup, row)* | 2 | 1 | 6 | 26 | 24 | 18 | 14 | 7 | 100 |
|  | *% (time interval, column)* | 37 | 29 | 33 | 43 | 47 | 45 | 43 | 41 | 43 |
| *4–9* | *N* | 845 | 699 | 2472 | 8109 | 6544 | 4984 | 4156 | 2326 | 30135 |
|  | *% (subgroup, row)* | 3 | 2 | 8 | 27 | 22 | 17 | 14 | 8 | 100 |
|  | *% (time interval, column)* | 34 | 36 | 35 | 33 | 32 | 32 | 32 | 32 | 33 |
| ≥10 | *N* | 720 | 682 | 2322 | 5815 | 4314 | 3510 | 3268 | 1896 | 22527 |
|  | *% (subgroup, row)* | 3 | 3 | 10 | 26 | 19 | 16 | 15 | 8 | 100 |
|  | *% (time interval, column)* | 29 | 35 | 33 | 24 | 21 | 23 | 25 | 26 | 25 |
| Total | *N* | 2473 | 1953 | 7120 | 24286 | 20399 | 15453 | 13020 | 7160 | 91864 |
|  | *% (subgroup, row)* | 3 | 2 | 8 | 26 | 22 | 17 | 14 | 8 | 100 |
|  | *% (time interval, column)* | 100 | 100 | 100 | 100 | 100 | 100 | 100 | 100 | 100 |
| Abbreviations: NIHSS, National Institutes of Health Stroke Scale; SU, stroke unit. | | | | | | | | | | |

| **Supplemental Table S2.** Diurnal admission profile of stroke patients stratified by NIHSS Score (multivariable Poisson regression analysis) | | | | | | | | |
| --- | --- | --- | --- | --- | --- | --- | --- | --- |
| **Hospital admission time** | **NIHSS Score ≤3** | |  | **NIHSS Score 4–9** | |  | **NIHSS Score ≥10** | |
|  | **RR (95% CI)** | **P value** |  | **RR (95% CI)** | **P value** |  | **RR (95% CI)** | **P value** |
| 0–3 h | ref. |  |  | ref. |  |  | ref. |  |
| >3–6 h | 0.63 (0.57, 0.70) | <0.0001 |  | 0.83 (0.75, 0.91) | 0.0002 |  | 0.95 (0.86, 1.05) | 0.31 |
| >6–9 h | 2.56 (2.37, 2.77) | <0.0001 |  | 2.92 (2.70, 3.15) | <0.0001 |  | 3.22 (2.98, 3.48) | <0.0001 |
| >9–12 h | 11.36 (10.71, 12.05) | <0.0001 |  | 9.58 (8.86, 10.36) | <0.0001 |  | 8.08 (7.48, 8.74) | <0.0001 |
| >12–15 h | 10.49 (9.89, 11.12) | <0.0001 |  | 7.77 (7.18, 8.40) | <0.0001 |  | 5.99 (5.54, 6.48) | <0.0001 |
| >15–18 h | 7.69 (7.11, 8.32) | <0.0001 |  | 5.87 (5.43, 6.35) | <0.0001 |  | 4.85 (4.49, 5.25) | <0.0001 |
| >18–21 h | 6.11 (5.65, 6.61) | <0.0001 |  | 4.90 (4.53, 5.30) | <0.0001 |  | 4.53 (4.19, 4.90) | <0.0001 |
| >21–23: 59 h | 3.22 (2.98, 3.48) | <0.0001 |  | 2.75 (2.54, 2.97) | <0.0001 |  | 2.64 (2.44, 2.85) | <0.0001 |
| Abbreviations: CI, confidence interval; NIHSS, National Institutes of Health Stroke Scale; ref., reference; RR, relative rate. | | | | | | | | |
